# A Curious Case of Pericardial Effusion Diagnosed as Diffuse Large B-cell Lymphoma

**DOI:** 10.7759/cureus.65991

**Published:** 2024-08-02

**Authors:** Madhulika L Mahashabde, Harin M Bhavsar, Yash R Bhimani

**Affiliations:** 1 General Medicine, Dr. D. Y. Patil Medical College, Hospital & Research Centre, Dr. D. Y. Patil Vidyapeeth, Pune, Pune, IND

**Keywords:** diffuse large b-cell lymphoma, non-hodgkin's lymphoma, pet-ct, r-chop regimen, pericardial effusion

## Abstract

Lymphoma arises from mature B, T, and natural killer (NK) cells. Lymphomas are classified into Hodgkin's lymphoma (HL) and non-Hodgkin's lymphoma (NHL). Diffuse large B-cell lymphoma (DLBCL) is a type of NHL. It can present with symptoms such as fever, chills, or night sweats, as well as symptoms due to extranodal involvement. Extranodal sites can include the gastrointestinal tract or renal involvement. A higher risk of developing diffuse large B-cell lymphoma (DLBCL) is seen in patients with congenital or acquired immunodeficiency, those on immunosuppression, and those with autoimmune disorders. In this case report, we present a case of pericardial effusion that, upon further evaluation, was diagnosed as diffuse large B-cell lymphoma (DLBCL). A 64-year-old male presented with complaints of retrosternal chest pain that progressed from New York Heart Association (NYHA) Grade II to IV over a month. The chest pain was moderate intensity, dull aching, and non-radiating. It was associated with orthopnea, paroxysmal nocturnal dyspnea, and anasarca. A chest X-ray (posteroanterior {PA} view) showed cardiomegaly with an increased cardiothoracic ratio, mediastinal widening, and pulmonary congestion. Echocardiography revealed moderate non-tappable pericardial effusion. A high-resolution computed tomography (HRCT) chest scan showed moderate pericardial effusion and a homogeneous enhancing mass in the left anterior superior mediastinum. A computed tomography (CT)-guided biopsy was performed to check for lymphoma, thymoma, or tuberculosis. The patient was diagnosed with diffuse large B-cell lymphoma (DLBCL). Owing to the diverse manifestations of diffuse large B-cell lymphoma (DLBCL), prompt diagnosis is required for controlling disease progression.

## Introduction

Lymphoma is the malignancy of lymphocytes within the lymphoid system, arising from B, T, or natural killer (NK) cells. It encompasses two main categories: non-Hodgkin's lymphoma (NHL) and Hodgkin's lymphoma (HL), with approximately 80% of cases classified as non-Hodgkin's lymphoma. B cells are versatile and capable of differentiating into various pathways, contributing to a spectrum of disorders with diverse clinical and pathological characteristics [1]. There are around 30 recognized subtypes of non-Hodgkin's lymphoma (NHL), with diffuse large B-cell lymphoma (DLBCL) being the most prevalent, comprising about 30% of all NHL cases [2]. The risk factors for DLBCL include immunosuppression, autoimmune disorders, and congenital and acquired immunodeficiency. The symptoms of lymphoma can include swollen lymph nodes in the neck or groin, fatigue, chest pain, cough, dyspnea if chest swelling occurs, back pain from bone involvement, and unusual bleeding or bruising. B symptoms, such as night sweats, fever, and weight loss, are common.

Extranodal involvement occurs in approximately 50% of patients, most frequently affecting the gastrointestinal tract and skin. Tumor lysis syndrome or lymphadenopathy can lead to ureteral obstruction and secondary renal involvement. Aggressive NHL may present with extranodal manifestations, including airway compression and superior vena cava syndrome. The symptoms related to infiltrated organs may develop if extranodal disease extends beyond the lymphatic system. The common sites of extranodal involvement include the skin, bones, spinal cord, and testicles [3]. Cardiac involvement as an initial sign of lymphoma is exceedingly rare, with DLBCL most commonly affecting the right atrium [4]. Primary cardiac lymphoma is confined to the heart and pericardium, whereas mediastinal DLBCL invades the heart [5]. B-cell lymphomas can also be associated with paraneoplastic syndromes. Treatment typically involves rituximab-based chemotherapy regimens, such as rituximab, cyclophosphamide, doxorubicin, vincristine, and prednisone (R-CHOP), or alternative combinations such as rituximab, polatuzumab vedotin, cyclophosphamide, doxorubicin, and prednisone (R-pola-CHP) [6,7].

## Case presentation

A 64-year-old male, a farmer by occupation, came with complaints of precordial chest pain and breathlessness that progressed from New York Heart Association (NYHA) Grade II to IV over a month, along with orthopnea, paroxysmal nocturnal dyspnea, and widespread edema that had been present for 12 days. The patient had retrosternal chest pain that was moderate in intensity, dull aching, and non-radiating. There was no associated history of fever, cold, palpitation, or diaphoresis. There was no history of addiction, smoking, drinking, or tobacco use. Additionally, there were no comorbidities reported, and there was no family history of malignancy. On examination, the patient had tachycardia and raised jugular venous pressure with mild pallor. There was no icterus, cyanosis, clubbing, edema, lymphadenopathy, or distended veins. On systemic examination, crepitations were present in the bilateral infra-axillary region and infra-scapular region, heart sounds were muffled but no additional heart sounds, and no third or fourth heart sounds were present. The patient's laboratory investigations on the day of admission are shown in Table 1.

**Table 1:** Laboratory investigations Alanine transaminase (ALT), aspartate transaminase (AST), and alkaline phosphatase (ALP) are enzymes that assess liver function. Total leukocyte count (TLC) measures the number of white blood cells in the blood. Cartridge-based nucleic acid amplification test (CBNAAT) is used for detecting nucleic acids of pathogens. Erythrocyte sedimentation rate (ESR) and C-reactive protein (CRP) are markers of inflammation. The units of measurement include gram per deciliter (g/dL), milligram per deciliter (mg/dL), microgram per deciliter (µg/dL), and units per liter (U/Lt) Hba1c, glycosylated hemoglobin; A/G, albumin/globulin; HBsAg, hepatitis B surface antigen; HCV, hepatitis C virus

Laboratory investigation	Report	Reference range
Hemoglobin	13 g/dL	13.2-16.6 g/dL
Total leukocyte count	7,000/µL	4,000-10,000/µL
Platelet	310,000/µL	150,000-410,000/µL
Urea	28 mg/dL	17-49 mg/dL
Creatinine	1.6 mg/dL	0.6-1.35 mg/dL
Total bilirubin	1.15 mg/dL	0.2-1.2 mg/dL
Direct bilirubin	0.48 mg/dL	0.5 mg/dL
AST	34 U/Lt	8-48 U/Lt
ALT	71 U/Lt	7-55 U/Lt
ALP	118 U/Lt	40-129 U/Lt
Hba1c	6.3%	<5.6%
Total protein	7.10 g/dL	5.5-8 g/dL
Serum albumin	4.2 g/dL	3.5–5 g/dL
A/G ratio	1.45	1.1-2.5
ESR	44 mm/hour	<20 mm/hour
CRP	15 mg/dL	<2 mg/dL
HIV	Negative	Negative
HBsAg	Negative	Negative
HCV	Negative	Negative
D-dimer	598 µg/L	<500 µg/L
CBNAAT	Negative	Negative

**Figure 1:**
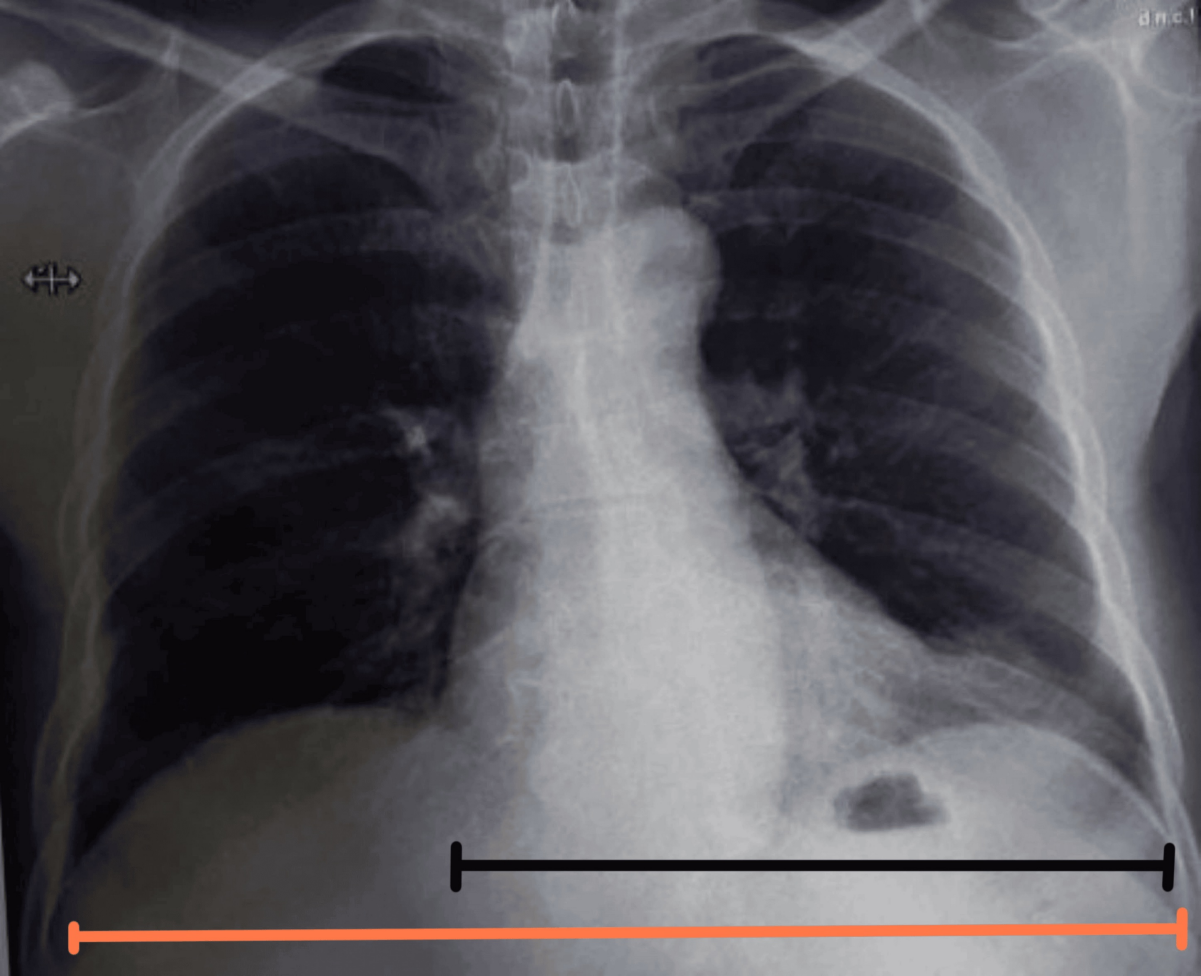
Chest X-ray posteroanterior view showing increased cardiothoracic ratio suggestive of cardiomegaly The cardiothoracic ratio is the ratio of the cardiac margin (black line) divided by the thoracic margin (orange line). A ratio of more than 0.5 in adults suggests cardiomegaly

**Figure 2:**
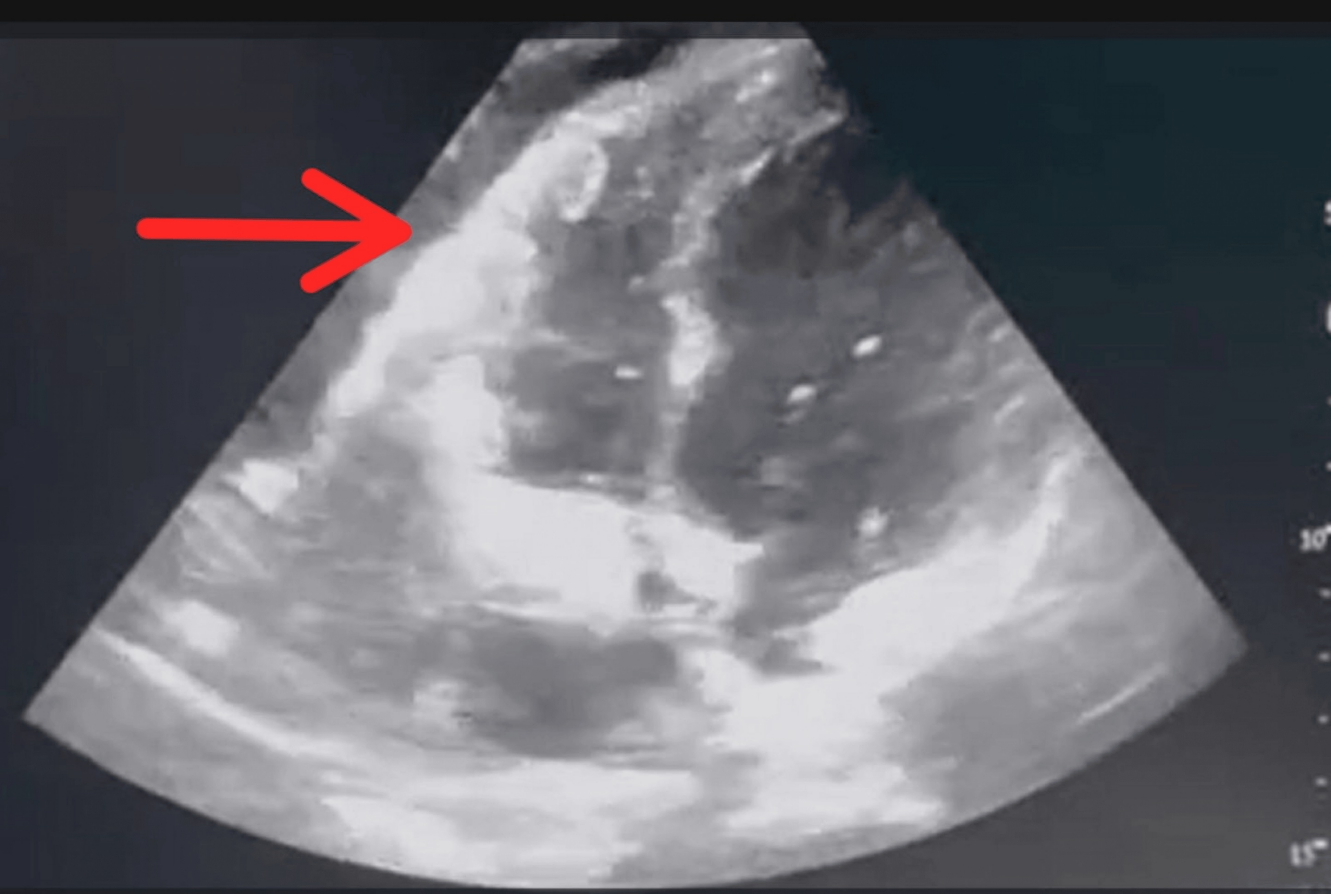
In apical four-chamber view on 2D echocardiography, moderate pericardial effusion can be seen (red arrow)

**Figure 3:**
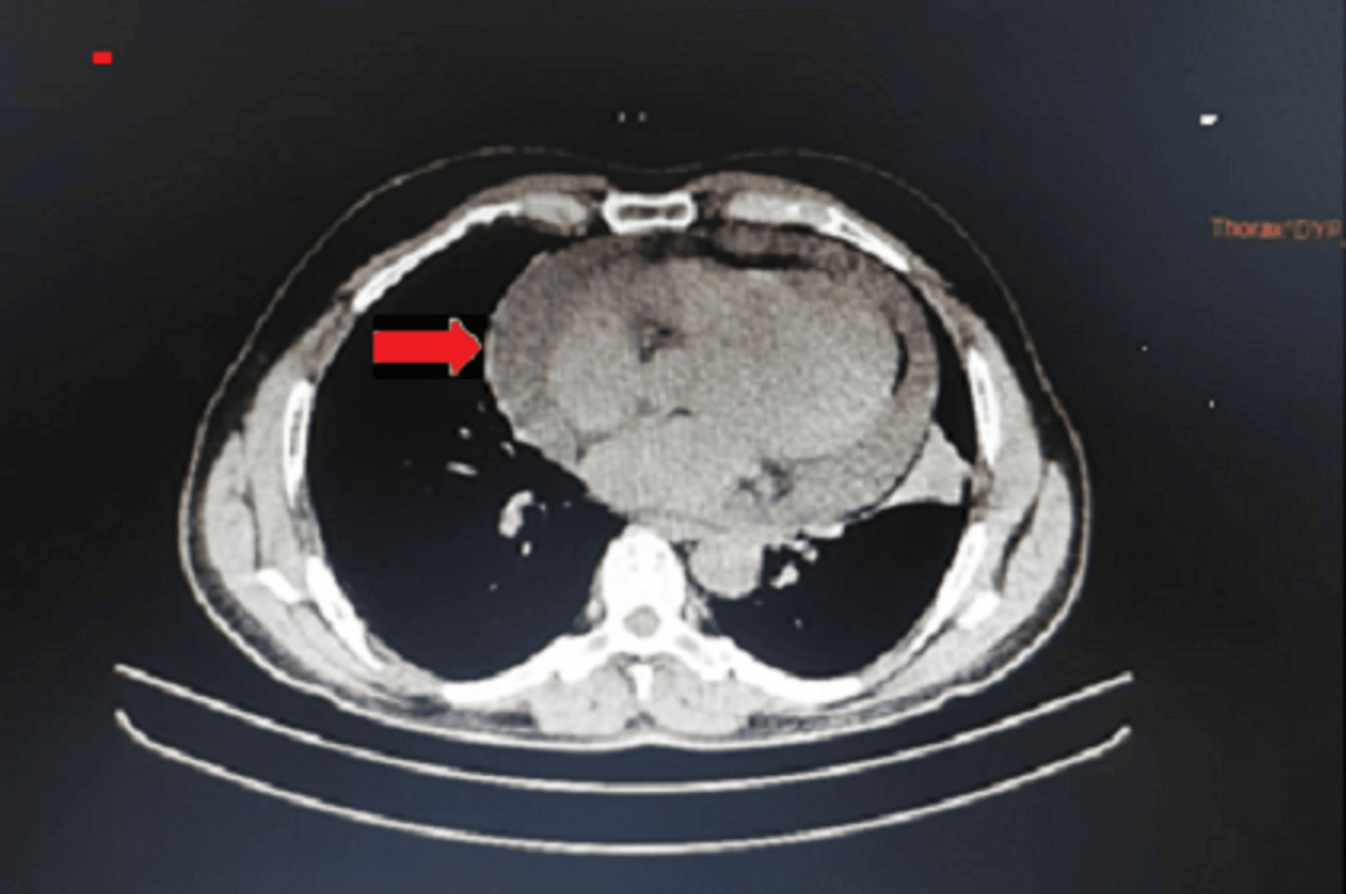
High-resolution computed tomography (HRCT) of the thorax revealed moderate to gross pericardial effusion with homogenously enhancing mass lesion

**Figure 4:**
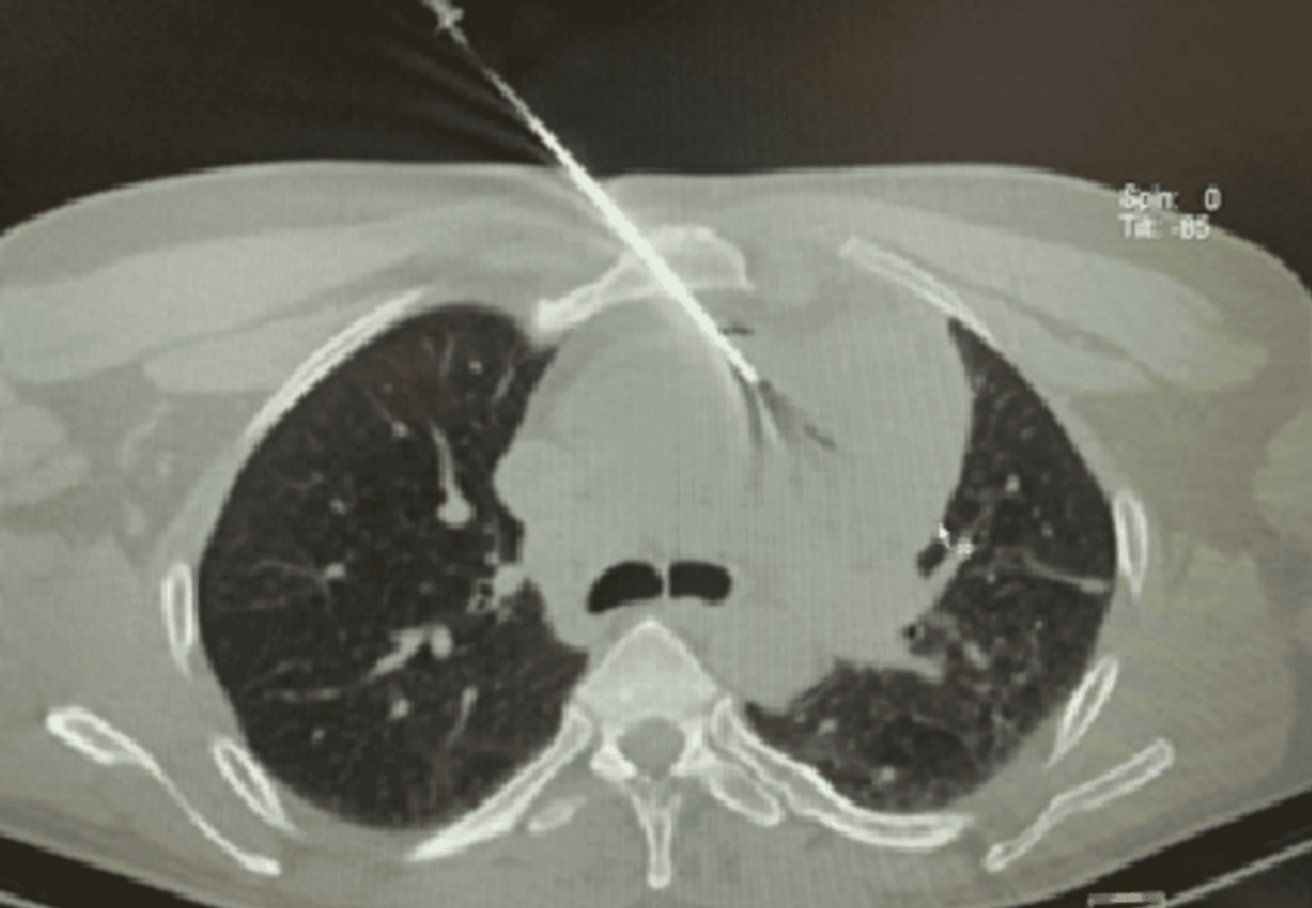
Computed tomography (CT)-guided transsternal biopsy of mediastinal mass

**Figure 5:**
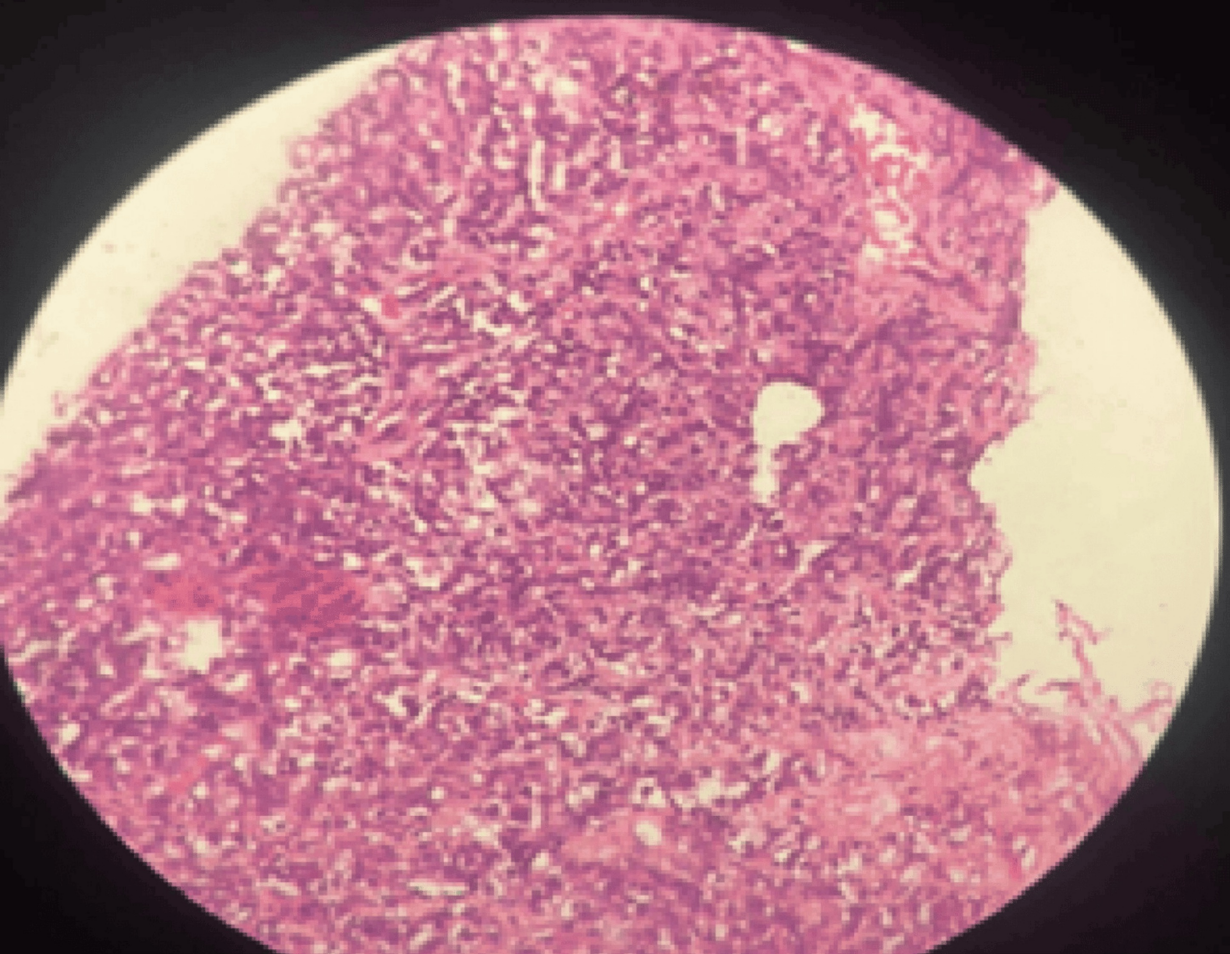
Histopathological analysis showed large cells with irregular nuclear margins and few scattered small lymphoid cells, with some of the cells showing large pleomorphic nuclei and pale cytoplasm, suggestive of diffuse large B-cell lymphoma (DLBCL)

Chest X-ray (posteroanterior {PA} view) showed cardiomegaly with an increased cardiothoracic ratio, mediastinal widening, and pulmonary congestion (Figure 1). Echocardiography revealed moderate pericardial effusion, with Grade I diastolic dysfunction (Figure 2). Therefore, high-resolution computed tomography (HRCT) of the thorax was performed, revealing moderate to extensive pericardial effusion. A homogenously enhancing mass lesion was observed with an epicenter in the left anterior-superior mediastinum, encasing the arch of the aorta. It extended inferiorly into the middle mediastinum, encasing the ascending aorta, main pulmonary trunk, right superior pulmonary vein, and right pulmonary artery. The mass was also seen extending to the inferior basal aspect of the heart, wrapping around the atrioventricular and interventricular groove (Figure 3). Therefore, a computed tomography (CT)-guided biopsy of the mediastinal mass was performed for further evaluation, and the sample was sent for histological analysis (Figure 4). On histopathological examination, there were large cells with irregular nuclear margins and a few scattered small lymphoid cells. Some of the cells showed large pleomorphic nuclei and pale cytoplasm, suggestive of diffuse large B-cell lymphoma (DLBCL). Immunohistochemistry was positive for cluster of differentiation (CD) 20 in large cells and CD3-positive in background cells, with 80% positivity for Ki-67 (Figure 5).

## Discussion

DLBCL is the most common type of non-Hodgkin's lymphoma. DLBCL comprises one-third of all NHL cases. The risk factors for DLBCL include immunosuppression, autoimmune disorders, and congenital and acquired immunodeficiency [8]. Tumor cells typically express B-cell antigens (CD19, CD20, and CD79a) and monoclonal IgM. The rearrangements of B-cell lymphoma 6 (BCL6) occur in approximately 30% of DLBCLs. About 20%-30% of DLBCLs are associated with the t(14;18) translocation. B symptoms occur in 30% of patients, and serum lactate dehydrogenase (LDH) levels are elevated in over half of them. Extranodal disease is present in 40% of cases and may affect the gastrointestinal tract, testis, bone, thyroid, skin, central nervous system, and/or bone marrow. Typically, heart involvement occurs later in the course of DLBCL. Dissemination occurs through the direct extension of the tumor, hematogenous spread, or lymphatic spread. The direct extension of the tumor leads to pericardial disease. The major involvement of the epicardial-adventitial region is believed to be caused by retrograde lymphatic spread. Hematogenous spread is responsible for the widespread interstitial-perivascular pattern, which is not specific to the heart's endocardial or epicardial elements [9].

For diagnosis, a CT scan is used when a patient's images from other noninvasive techniques are insufficient when they are known to be contraindicated for cardiac magnetic resonance imaging (CMRI) [10]. Heart lymphoma typically presents as hypo-attenuating masses on CT. These masses extend along the epicardium surrounding the coronary arteries and infiltrate the myocardium. The diagnosis of cardiac lymphoma using 18F-fluorodeoxyglucose positron emission tomography (18F-FDG PET) alone presents difficulties as it has low resolution and the radiotracer physiologically accumulates more in the myocardium [11]. The patients with cardiac DLBCL have demonstrated good response rates to chemotherapy using R-CHOP. In one case series, the survival rate was 30 months, and the overall response rate of primary cardiac lymphoma to this treatment was 79% [6]. Alternatives include R-pola-CHP, which, compared to R-CHOP, showed a higher survival rate without progression at two years [7].

In addition to chemotherapy, surgical resection may be undertaken in the early stages of the disease [12,13]. Debulking surgery becomes necessary for patients presenting with hemodynamic impairment and superior vena cava syndrome. Surgery may serve as a palliative intervention in cases of severe right ventricular outflow obstruction to increase lung blood flow. Patients experiencing worsening illness even after chemotherapy may benefit from radiation therapy [14]. Prognosis in cardiac DLBCL varies based on the disease stage, extent of cardiac involvement, and response to treatments such as R-CHOP.

## Conclusions

Diagnosing cardiac DLBCL typically requires multimodal imaging because the condition can present with subtle symptoms. Physicians should consider lymphoma in the differential diagnosis when evaluating a cardiac tumor. It is important to distinguish mediastinal DLBCL with cardiac involvement from primary cardiac lymphoma due to differences in prognosis. Given the aggressive nature of DLBCL and its rapid growth, successful treatment relies on early detection and conventional chemotherapy.
